# Compared Efficacy of Adjuvant Intravesical BCG-TICE vs. BCG-RIVM for High-Risk Non-Muscle Invasive Bladder Cancer (NMIBC): A Propensity Score Matched Analysis

**DOI:** 10.3390/cancers14040887

**Published:** 2022-02-10

**Authors:** Francesco Del Giudice, Rocco Simone Flammia, Benjamin I. Chung, Marco Moschini, Benjamin Pradere, Andrea Mari, Francesco Soria, Simone Albisinni, Wojciech Krajewski, Tomasz Szydełko, Ekaterina Laukhtina, David D’Andrea, Andrea Gallioli, Laura S. Mertens, Martina Maggi, Alessandro Sciarra, Stefano Salciccia, Matteo Ferro, Carlo Maria Scornajenghi, Vincenzo Asero, Susanna Cattarino, Mario De Angelis, Giovanni E. Cacciamani, Riccardo Autorino, Savio Domenico Pandolfo, Ugo Giovanni Falagario, Nicola D’Altilia, Vito Mancini, Marco Chirico, Francesco Cinelli, Carlo Bettocchi, Luigi Cormio, Giuseppe Carrieri, Ettore De Berardinis, Gian Maria Busetto, on behalf of European Association of Urology (EAU)—Young Academic Urologists (YAU) Urothelial Cancer Working Party

**Affiliations:** 1 Department of Maternal-Infant and Urological Sciences, Policlinico Umberto I Hospital, “Sapienza” Rome University, 00185 Rome, Italy; roccosimone92@gmail.com (R.S.F.); martina.maggi@uniroma1.it (M.M.); alessandro.sciarra@uniroma1.it (A.S.); stefano.salciccia@uniroma1.it (S.S.); carlomaria.scornajenghi@uniroma1.it (C.M.S.); vincenzo.asero@uniroma1.it (V.A.); susanna.cattarino@uniroma1.it (S.C.); ettore.deberardinis@uniroma1.it (E.D.B.); 2 Department of Urology, Stanford Medical Center, Stanford, CA 94305, USA; bichung@stanford.edu; 3 Department of Urology, San Raffaele Hospital and Scientific Institute, 20132 Milan, Italy; marco.moschini87@gmail.com (M.M.); deangelis.mario@hsr.it (M.D.A.); 4 Department of Urology, Medical University of Vienna, 1090 Vienna, Austria; benjaminpradere@meduniwien.ac.at (B.P.); katyalaukhtina@gmail.com (E.L.); dd.dandrea@gmail.com (D.D.); 5 Unit of Oncologic Minimally-Invasive Urology and Andrology, Department of Experimental and Clinical Medicine, Careggi Hospital, University of Florence, 50134 Florence, Italy; andreamari08@gmail.com; 6 Urology Division, Department of Surgical Sciences, University of Studies of Torino, 10126 Turin, Italy; soria.fra@gmail.com; 7 Urology Department, Erasme Hospital, Université Libre de Bruxelles, 1070 Brussels, Belgium; albisinni.simone@gmail.com; 8 Department of Urology and Oncological Urology, Wrocław Medical University, 50-556 Wrocław, Poland; krajwoj@gmail.com (W.K.); tomasz.szydelko1@gmail.com (T.S.); 9 Institute for Urology and Reproductive Health, Sechenov University, 19435 Moscow, Russia; 10 Unit of Urology, Fundacio Puigvert, 08025 Barcelona, Spain; andrea.gallioli@gmail.com; 11 Department of Urology, The Netherlands Cancer Institute, 1066 Amsterdam, The Netherlands; ls.mertens@gmail.com; 12 Division of Urology, European Institute of Oncology (IEO)-IRCCS, 20141 Milan, Italy; matteo.ferro@ieo.it; 13 USC Institute of Urology, University of Southern California, Los Angeles, CA 90089, USA; giovanni.cacciamani@med.usc.edu; 14 Division of Urology, Department of Surgery, Virginia Commonwealth University, Richmond, VA 23284, USA; ricautor@gmail.com (R.A.); pandolfosavio@gmail.com (S.D.P.); 15 Department of Neurosciences, Sciences of Reproduction, and Odontostomatology, Fderico II University, 80131 Naples, Italy; 16 Department of Urology and Renal Transplantation, Policlinico Riuniti di Foggia, University of Foggia, 71122 Foggia, Italy; ugofalagario@gmail.com (U.G.F.); dattilanicola@gmail.com (N.D.); mancini.uro@gmail.com (V.M.); chirico.marco90@gmail.com (M.C.); francescocinelli90@gmail.com (F.C.); carlo.bettocchi@unifg.it (C.B.); luigi.cormio@unifg.it (L.C.); giuseppe.carrieri@unifg.it (G.C.); gianmaria.busetto@unifg.it (G.M.B.)

**Keywords:** bladder cancer, re-TUR, BCG strain, BCG-TICE, BCG-RIVM, recurrence-free survival, progression-free survival, cancer-specific survival

## Abstract

**Simple Summary:**

Intravesical immunotherapy with bacillus Calmette–Guerin (BCG) is the standard therapy for high-risk non-muscle invasive bladder cancer. Different BCG strains are currently available and the superiority of any BCG strain over another could not be demonstrated yet. We compared the efficacy of two BCG strains: RIVM and TICE, respectively. In this propensity-score matched cohort study, we showed no particular survival benefit of TICE vs RIVM in the case of high-risk disease. Nevertheless, stratifying our data for re-staging procedures and for those who received BCG maintenance, we identified BCG TICE to improve RFS independently. Herein, we corroborated the importance of performing a routine secondary resection followed by an adequate maintenance course of BCG. Future larger prospective randomized head-to-head trials are needed to further elucidate this important topic, especially in this era of BCG shortage.

**Abstract:**

Background: Intravesical immunotherapy with bacillus Calmette–Guerin (BCG) is the standard therapy for high-risk non-muscle invasive bladder cancer (NMIBC). The superiority of any BCG strain over another could not be demonstrated yet. Methods: Patients with NMIBCs underwent adjuvant induction ± maintenance schedule of intravesical immunotherapy with either BCG TICE or RIVM at two high-volume tertiary institutions. Only BCG-naïve patients and those treated with the same strain over the course of follow-up were included. One-to-one (1:1) propensity score matching (PSM) between the two cohorts was utilized to adjust for baseline demographic and tumor characteristics imbalances. Kaplan–Meier estimates and multivariable Cox regression models according to high-risk NMIBC prognostic factors were implemented to address survival differences between the strains. Sub-group analysis modeling of the influence of routine secondary resection (re-TUR) in the setting of the sole maintenance adjuvant schedule for the two strains was further performed. Results: 852 Ta-T1 NMIBCs (*n* = 719, 84.4% on TICE; *n* = 133, 15.6% on RIVM) with a median of 53 (24–77) months of follow-up were reviewed. After PSM, no differences at 5-years RFS, PFS, and CSS at both Kaplan–Meier and Cox regression analyses were detected for the whole cohort. In the sub-group setting of full adherence to European/American Urology Guidelines (EAU/NCCN), BCG TICE demonstrated longer 5-years RFS compared to RIVM (68% vs. 43%, *p* = 0.008; HR: 0.45 95% CI 0.25–0.81). Conclusion: When routinely performing re-TUR followed by a maintenance BCG schedule, TICE was superior to RIVM for RFS outcomes. However, no significant differences were detected for PFS and CSS, respectively.

## 1. Introduction

Adjuvant intravesical Bacillus of Calmette–Guérin (BCG) for the treatment of non-muscle invasive bladder cancer (NMIBC) was successfully introduced in 1976 by Morales et al. [1]. It was subsequently established as the standard of care following trans-urethral resection of bladder tumor (TURBT) for intermediate/high-risk cases according to both American and European Urology Guidelines (National Comprehensive Cancer Network [NCCN], EAU) [2,3,4]. For optimal efficacy, the induction course of BCG should be followed by at least one year of maintenance treatment [2]. In particular, evidence from randomized controlled trials (RCT) revealed that the three-year BCG maintenance protocol originally described by Lamm et al. [5] for high-risk NMIBCs, is associated with prolonged recurrence-free rates. However, there was no difference in progression and survival outcomes compared with the one-year-only maintenance schedule. Also, there were no differences for intermediate-risk tumors between different maintenance schedules [6,7]. The recently released NIMBUS trial [8] demonstrated that a reduced-frequency BCG schedule, or the use of dose-reduced instillations, are inferior to the standard schedule regarding the time to first recurrence. These considerations, together with the above-mentioned studies, are relevant in light of recent supply chain issues in acquiring BCG. Moreover, alternative treatments such as device-assisted chemotherapy instillations (microwave-induced hyperthermia [RITE] or electromotive drug administration [EMDA]) are experimental, less effective, and significantly more expensive, further highlighting the issues of BCG shortages. For these reasons, international panels provided recommendations on rationing BCG by updating Guidelines indications and suggesting alternative strategies.

Currently, evidence supporting differences in the efficacy of different BCG strains is lacking, and, based on meta-analyses and post-hoc RCTs, EAU guidelines state that there seem to be no clear differences between the different BCG strains [9,10,11]. However, lack of granularity in reporting data on adoption of secondary resection (re-TUR) or uniform utilization of maintenance schedule is a major limitation in data interpretation. Recently, our group demonstrated in a retrospective single-institution study the importance of strictly adhering to EAU Guidelines by routinely performing re-TUR and that, in this setting, BCG TICE administration was independently associated with prolonged RFS when compared to BCG RIVM or BCG Connaught [12].

Given these findings, in this current study, our first aim is to compare the most widely used American and European BCG strains (i.e., TICE and RIVM) at two Italian tertiary centers to elucidate which BCG strain is most effective in a large cohort of high-risk NMIBCs. Additionally, we seek to assess the weighted influence of routine secondary resection and adopting maintenance schedule on survival differences per the specific BCG strain utilized.

## 2. Patients and Methods

### 2.1. Eligibility CRITERIA

This is a dual-institution retrospective cohort study of patients diagnosed with NMIBC who received an adjuvant induction intravesical immunotherapy with two different BCG strains, with or without a BCG-maintenance schedule, over a period of 14 years.

According to risk classification criteria currently available within EAU/NCCN guidelines, we consecutively reviewed patients between 2004 and 2018 who were defined as high-risk NMIBC and were considered eligible as long as they were BCG-naïve patients. Data were also collected on available patients who underwent re-staging procedures (re-TUR), defined as within the time frame of 2 to 6 weeks following primary resection.

Patients with a history of muscle-invasive disease (T2 or higher), upper tract urothelial cancer (UTUC), non-urothelial carcinoma, those who previously received BCG (non-naïve patients), or those who did not receive BCG as an initial intravesical treatment for a high-risk NMIBC were considered ineligible. Patients for whom incomplete/missing data precluded obtaining all the requested information were also excluded from the final analysis.

### 2.2. BCG Protocol, Treatment Schedule, and Strains Administered

The adjuvant BCG immunotherapy protocol was started 2 to 3 weeks after staging TUR or Re-TUR. The induction course consisted of one instillation every week for 6 consecutive weeks followed by 3 years of maintenance instillation with 3 weekly instillations every 3 months for the first two schedules and then every 6 months, as originally described by Lamm et al. [13]. A full BCG course consisted of 27 instillations, divided into an induction course and 7 maintenance courses. Any instillation beyond the 6 of the induction course was defined as maintenance BCG.

Patients were only reviewed if they were treated with the same BCG strain over the course of the follow-up. The two BCG strains available over the range of study time were: (1) BCG seed TICE (OncoTICE^®^, MSD, NJ, USA; 2–8 × 10^8^ CFU), and (2) BCG seed RIVM (Medac^®^, D-20354 Hamburg, Germany; 2 × 10^8^–3 × 10^9^ CFU).

Allocation of each strain to each patient was caused by differences in supply and distribution. Allocation was not planned and randomized; the choice of BCG selection over the years was made based on competitive price as well as on the commitment by the manufacturing company to ensure a steady supply of the intravesical therapy.

### 2.3. Statistical Analysis

Descriptive statistics were used to summarize pertinent study information. The association between variables was tested by the Pearson chi-square test or the Fisher’s exact test when appropriate.

One-to-one (1:1) propensity score matching (PSM) between the two cohorts was adopted to adjust for baseline demographic and tumor characteristic imbalances.

The univariate effect of BCG strain on survival outcomes was explored by the Kaplan–Meier product-limit method. The log-rank test was used to assess differences between the treatment arm. The risk of survival was expressed as hazard ratios (HR) and 95% confidence intervals (CI). Study endpoints were as follows: (I) determination of recurrence-free survival (RFS), defined as the months for BCa of any stage/grade to relapse; (II) progression-free survival (PFS), defined as the months for a rise to T2 or the higher stage; (III) duration of cancer-specific survival (CSS). Times to events were calculated by taking the date of starting BCG as time zero. Patients without an event were censored at the last date of follow-up.

As the two strains were not assigned by randomization, Cox proportional hazards multivariable regression analysis was performed to adjust for the number of prognostic factors previously determined by Gontero et al. [14] in the case of high-risk disease. In particular, the proportional hazards model was developed by adjusting for age (<70 vs. ≥70 years) for progression and survival, tumor focality (unifocal vs. multifocal) for recurrence, tumor dimension (<3 cm, ≥3 cm) for recurrence, progression and survival, and concomitant CIS (absence and presence) for progression. Moreover, adjustment for the use of re-TUR procedures and/or maintenance schedule administration, and separate analyses in patients who received it or not, were performed to verify and compare the benefit in survival outcomes among the different two strains according to the currently available pathway for high-risk NMBC patients. Finally, we identified the subgroup of patients who received both re-TUR plus BCG maintenance. In those patients, 1:1 PSM was performed to adjust for baseline demographic and tumor characteristics imbalances, and Cox proportional hazards multivariable regression analysis were refitted. Statistical Analyses were performed using the R software v.3.5.4 (R Core Team 2018, R: A language and environment for statistical computing. R Foundation for Statistical Computing, Vienna, Austria. URL https://www.R-project.org/, accessed on 5 January 2022), and all tests were two-sided with a significance level set at *p* < 0.05.

## 3. Results

Overall, 852 patients with a median age of 71 years (IQR: 64–79) were analyzed, *n* = 133 (15.6%) on TICE and *n* = 719 (84.4%) on RIVM. Demographics and tumor characteristics of the whole unmatched cohort are presented in Supplementary Table S1. The vast majority of the patients were primary/recurrent HG Ta-T1 (*n* = 829, 97.4%). Out of these, the most represented were primary and recurrent HG T1 with *n* = 349, 41.0%, and *n* = 219, 25.7%, cases, respectively (Supplementary Table S1).

Given the existence of substantial heterogeneity among the two cohorts in terms of age, gender, and other NMIBC prognostic risk factors, a PSM was applied across the whole study population. Specifically, one-to-one (1:1) PSM for age, gender, T stage, tumor grade, focality, size, concomitant CIS, adoption of re-TUR and BCG maintenance schedule resulted in two equally distributed groups of *n* = 133 BCG TICE and RIVM respectively with no residual statistical imbalances (Table 1).

The median number of BCG instillations administered was 17 (IQR: 16–20) and 18 (IQR: 16–20) in the TICE and RIVM group, respectively (*p* = 0.73). Additional information regarding tolerability profile and drop-off due to side effects according to the two strains are summarized in Supplementary Table S2, with no clinically relevant nor significant differences detected among the two groups.

### 3.1. Recurrence-Free Survival (RFS)

The median follow-up for the whole cohort was 53 months (IQR: 24–77). Overall, *n* = 46 (34.6%) patients on TICE, and *n* = 290 (40.3%) on RIVM recurred. Recurrence rates, stratified by re-TUR (yes vs. no), were 25.3% and 46.6% for patients who received TICE, and 35.9% and 45.0% for those with RIVM. Similarly, for maintenance BCG course (yes vs. no), were 23.1% vs. 35.8% on TICE and 35.8% vs. 43.3% on RIVM, respectively.

After adjustment for the risk of recurrence, undergoing re-TUR was found as an independent predictor for prolonged RFS (HR: 0.71; 95% CI: 0.57–0.88; *p* = 0.002) with no differences detected when after the procedure was administered one of the two strain (re-TUR HR_TICE_: 0.54; 95% CI: 0.30–0.97; *p* = 0.040; re-TUR HR_RIVM_: 0.74, 95% CI: 0.59–0.94, *p* = 0.012). The same trend was observed for the utilization of a maintenance schedule, which independently prolonged RFS (HR: 0.57; 95% CI: 0.46–0.71; *p* < 0.001), with no differences detected within the two-strain related effect (HR_TICE_: 0.21; 95% CI: 0.06–0.79; *p* = 0.021; HR_RIVM_: 0.58, 95% CI: 0.46–0.74, *p* < 0.001).

**Figure 1:**
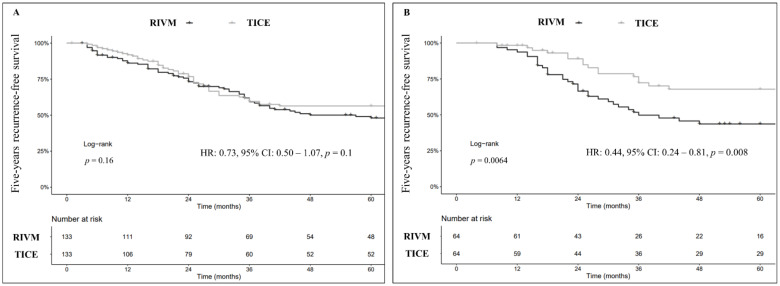
Kaplan–Meier curves (Log-rank) depicting the effect of BCG strains (TICE vs. RIVM) on 5-years recurrence-free survival (RFS) in the study cohort after 1:1 PSM (*n* = 266) (**A**) and in the sole sub-group of patients who had secondary resection (re-TUR) followed by BCG induction plus maintenance after 1:1 PSM (*n* = 128) (**B**).

After 1:1 PSM, RFS at 5-yr was 56% (95% CI: 48–67%), and 48% (95% CI: 40–58%) for TICE and RIVM respectively (Log-rank, *p* = 0.2; Figure 1A). Modeling the whole PSM cohort for recurrence prognostic factors revealed no significant difference in RFS outcomes (HR: 0.73, 95% CI: 0.50–1.07, *p* = 0.1).

More interestingly, in the sub-analysis of the sole re-TUR followed by BCG maintenance patients (*n* = 128), TICE was associated with prolonged time to first recurrence compared to RIVM (HR: 0.44, 95% CI: 0.24–0.81, *p* = 0.008; Figure 1B).

### 3.2. Progression-Free Survival (PFS)

Overall, 23 (17.3%) patients on TICE and 158 (22.0%) on RIVM progressed to MIBC. Progression rates, stratified by re-TUR, were 10.7 vs. 25.9% on TICE, and 18.4 vs. 25.8% on RIVM, while for maintenance BCG course were 18.3 vs. 7.7% on TICE and 15.8 vs. 26.0% on RIVM.

Re-TUR and maintenance protocol confirmed to independently improve PFS (HR: 0.72; 95% CI: 0.54–0.97, *p* = 0.03, and, HR: 0.51; 95% CI: 0.37–0.72, *p* < 0.001, respectively). No differences were detected within the two-strain related effect for re-TURB (HR_TICE_: 0.56; 95% CI: 0.23–1.37; *p* = 0.2; HR_RIVM_: 0.76, 95% CI: 0.56–1.05, *p* = 0.1) and maintenance BCG course (HR_TICE_ 0.29; 95% CI: 0.03–2.56; *p* = 0.3; HR_RIVM_ 0.51, 95% CI: 0.35–0.73, *p* < 0.001).

**Figure 2:**
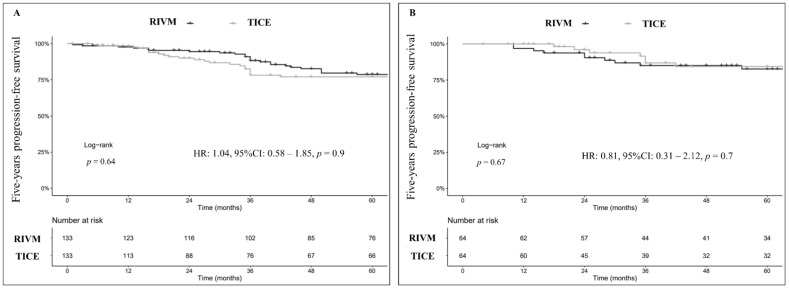
Kaplan–Meier curves (Log-rank) depicting the effect of BCG strains (TICE vs. RIVM) on 5-years progression-free survival (PFS) in the whole study cohort after 1:1 PSM (*n* = 266) (**A**) and in the sole sub-group of patients who had secondary resection (re-TUR) followed by BCG induction plus maintenance after 1:1 PSM (*n* = 128) (**B**).

After 1:1 PSM, PFS at 5-yr was 77% (95% CI: 69–86%) for TICE and 79% (95% CI: 72–87%) for RIVM, respectively (Log-rank, *p* = 0.6) (Figure 2A). When adjusting for progression predictors, no specific BCG strain showed an influence on time to progression (HR: 1.04, 95% CI: 0.58–1.85, *p* = 0.9).

In the sub-analysis of the sole re-TUR followed by BCG maintenance patients, there were no differences among the two stains for PFS (HR: 0.79, 95% CI: 0.30–2.10, *p* = 0.6; Figure 2B).

### 3.3. Cancer-Specific Survival (CSS)

Overall, *n* = 5 (3.8%) on TICE and *n* = 67 (9.3%) on RIVM died due to BCa. CSM rates, stratified by re-TUR, were 1.3 vs. 6.9% on TICE, and 7.3 vs. 11.5% on RIVM while for maintenance BCG course (yes vs. no) were 4.2 vs. 7.7% on TICE, and 5.6 vs. 26.0% on RIVM.

Although a protective effect was noted, re-TUR was not independently associated with prolonged time to BCa death (HR: 0.65, 95% CI: 0.40–1.04, *p* = 0.07). Conversely, the adoption of BCG maintenance offered a protective prolonging CSS (HR: 0.47, 95% CI: 0.27–0.83, *p* = 0.008).

**Figure 3:**
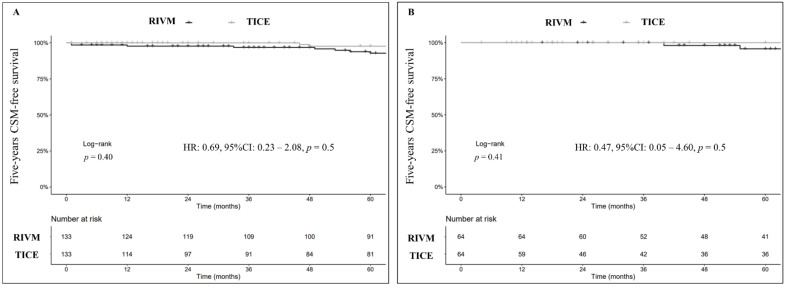
Kaplan–Meier curves (Log-rank) depicting the effect of BCG strains (TICE vs. RIVM) on 5-years cancer-specific survival (CSS) in the whole study cohort after 1:1 PSM (*n* = 266) (**A**) and in the sole sub-group of patients who had secondary resection (re-TUR) followed by BCG induction plus maintenance after 1:1 PSM (*n* = 128) (**B**).

After 1:1 PSM, Five-year CSS was 98% (95% CI: 95–99%) for TICE and 93% (95% CI: 88–98%) for RIVM (Log-rank, *p* = 0.4; Figure 3A). Similarly, to PFS, none of the two strains was independently associated with improved CSS both at whole cohort and sub-group analysis level (HR: 0.69, 95% CI: 0.23–2.08, *p* = 0.5; HR: 0.47, 95% CI: 0.05–4.60, *p* = 0.5, respectively; Figure 3B).

## 4. Discussion

Adjuvant intravesical BCG immunotherapy is the most effective organ-sparing treatment for intermediate/high-risk NMIBCs after TURBT ± re-TUR [2,3,4]. The mechanism through which BCG induces its beneficial effect is still not clear. Recent evidence shows that BCG creates an inflammatory response with a microenvironment that leads to increased production of pro-inflammatory cytokines and chemokines in the urothelium. Through this pro-inflammatory cytokine production, an antitumor immune response is mounted by trained immunity and the adaptive immune system [15]. As a consequence, BCG significantly reduces the recurrence rate and has an impact on early progression when the induction course is followed by at least one year of maintenance [5].

Genomic changes over the past 40–50 years have led to several worldwide available sub-strains, which ultimately may have altered or imbalanced the compared strain’s potential efficacy [16]. Additionally, culturing BCG strains is a tedious pharmaceutical process that takes over approximately three months to complete due to the slow doubling times of the mycobacterium. For this reason, worldwide shortages of BCG have occurred due to supply-chain limitations and production issues, especially after 2012 when the production of the BCG Connaught strain was suspended. Although the implications from international BCG shortages are potentially influencing survival outcomes for NMIBC patients, no clear recommendations are currently available on optimal BCG strain efficacy.

A recent meta-analysis from Huang et al. in 2017 identified ten different BCG strains in the published literature but could not confirm the superiority of any BCG strain; however, the study design was not specified to examine this question [17]. The same year, Bohem et al. showed how BCG strains exhibited a superior efficacy in terms of preventing recurrence compared to chemotherapy. Otherwise, no definitive conclusion was reached in this systematic review about the superiority of a specific BCG strain [18]. In addition, several other trials comparing different available BCG strains have been performed [19,20,21,22], but heterogeneity both in study design and the induction therapy protocols have not allowed for a consensus in strain superiority. Recently, Del Giudice et al. [12] published a historical retrospective cohort series comparing the three most frequently adopted strains, including Connaught, TICE, and RIVM. While the comprehensive comparison of the whole cohort did not highlight any substantial survival influence of one particular strain, there were differences in the sub-group analysis.

According to the EAU guidelines recommendations, those high-risk cases who undergo re-TUR and maintenance schedule demonstrate prolonged RFS when receiving BCG TICE. Interestingly, in the present study, after propensity score matching, we confirmed TICE to provide a longer time to first recurrence when compared to RIVM in a post-re-TUR and maintenance schedule setting (HR: 0.44, 95% CI: 0.24–0.81).

A second relevant point of discussion of the present analysis is the confirmation of independently improved recurrence and progression rates for those cases who received a re-TUR and a number of BCG instillations exceeding the induction course. We reported that the tolerability profile of the two strains was overlapping and not influencing the drop-off rate over the course of treatment.

The phenomenon for which TICE performs better than other strains in the setting of a maintenance schedule was previously documented by Witjes et al. in their cohort of HG T1 patients assessing the comparison with the no longer available BCG Connaught [23]. Such an improved performance of BCG Connaught in a no-maintenance schedule could be explained because the Connaught strain creates a more robust immune response earlier on during induction therapy course but might lose some of its efficacy during maintenance due to a decrease in the immune response over time. By contrast, TICE has long-acting immunogenic proprieties following the induction phase, supporting the idea that it needs a long-term duration therapy to exhibit its proper therapeutic potential. This was also demonstrated by Rentsch et al. [20], who showed how treatment with the BCG Connaught conferred significantly greater clinical 5-yr RFS compared with treatment with BCG TICE (*p* = 0.0108) but only in the setting of the sole induction course of treatment for high-risk NMIBC.

In their original series, Krajewski et al. [24] reported similar findings with the current analysis concerning both TICE and RIVM (5yr-RFS_TICE_: 61.1% and RFS_RIVM_: 60.5%). More interestingly, in a recently released multicentric comparative series of three strains (Moreau, TICE, and RIVM), Nowak et al. were able to identify how RIVM strain was significantly associated with inferior RFS compared to the Moreau (hazard ratio [HR] 1.69 for RIVM; *p* = 0.034) and TICE (HR 1.87 for RIVM; *p* = 0.002) strains [25]; however, no significant differences in PFS were observed among any of the strains.

D’Andrea et al. [26] published a previous cohort study, which analyzed a retrospective head-to-head cohort of patients allocated to BCG TICE and Moreau, respectively. Similar to the current report, the authors confirmed once again the importance of using BCG in a maintenance schedule (HR_RFS_, 0.32, *p* < 0.01; HR_PFS_, 0.28, *p* < 0.01) independently of the type of strain adopted (HR_RFS_, 0.87, *p* = 0.59 and HR_PFS_, 0.68, *p* = 0.36), and documented no significant difference in terms of RFS or PFS between TICE and Moreau (HR: 0.88, *p* = 0.58 and HR: 0.55, *p* = 0.14 respectively).

Except for these last aforementioned studies, there are limitations to be acknowledged among previous studies reporting differences between various BCG strains, including small sample size, absence of maintenance treatment assessment, and lack of information regarding secondary resection. To our knowledge, the present study represents the first propensity-matched analysis which, in lieu of significantly redesignating our original sample size, addresses survival outcomes on two perfectly matched cohorts for the aim of interest represented by the type of BCG strain adopted. For these reasons, our findings corroborate the evidence that, when treated according to EAU Guidelines, NMIBCs submitted to re-TUR followed by BCG with maintenance might achieve RFS benefit in the setting of the use of BCG TICE, highlighting possible future perspectives when re-staging procedures and long-term adjuvant schedules are performed.

Our study is not without limitations. First, these patients were accrued from a long-term retrospective cohort with all the inherent limitations to retrospective studies. Also, the distribution of the two different drugs was not randomized but was determined based on a cost-benefit analysis performed annually by each Institution with the assumption that there was no net benefit of one strain over the other. In addition, even though we adjusted our different survival outcomes for the most well-established prognostic risk estimators for high-risk NMIBC, there may be selection biases between the groups that we could not account for. Additionally, the data was from two high-volume academic tertiary institutions, which may limit generalizability.

## 5. Conclusions

In this propensity-score matched cohort study, we corroborated the importance of performing a routine secondary resection followed by an adequate maintenance course of BCG. After comparing two of the most widely adopted BCG strains, we showed no particular survival benefit of both TICE and RIVM in the case of high-risk disease. Nevertheless, stratifying our data for re-staging procedures and for those who received BCG in the setting of receiving maintenance, we identified BCG TICE to improve RFS independently. Future larger prospective randomized head-to-head trials are needed to further elucidate this important topic, especially in this era of BCG shortage.

## Figures and Tables

**Table 1:** Baseline demographic and clinic-pathologic characteristics of the propensity-matched study population according to BCG strain.

Characteristic	Overall, *n* = 266 ^1^	RIVM, *n* = 133 (50%) ^1^	TICE, *n* = 133 (50%) ^1^	*p*-Value ^2^
**Age**, years (IQR)	68 (64–74)	68 (64–75)	68 (62–73)	0.2
**Gender**				0.7
male	201 (75.6%)	99 (74.4%)	102 (76.7%)	
female	65 (24.4%)	34 (25.6%)	31 (23.3%)	
**Smoking Status**				0.5
No smoker	105 (39.5%)	57 (42.9%)	48 (36.1%)	
Former smoker	73 (27.4%)	33 (24.8%)	40 (30.1%)	
Active smoker	88 (33.1%)	43 (32.3%)	45 (33.8%)	
**Tumor Size**				>0.9
<3 cm	161 (60.5%)	80 (60.2%)	81 (60.9%)	
≥3 cm	105 (39.5%)	53 (39.8%)	52 (39.1%)	
**Tumor focality**				0.5
Unifocal	101 (38.0%)	53 (38.8%)	48 (36.1%)	
Multifocal	165 (62.0%)	80 (60.2%)	85 (63.9%)	
**T stage**				0.9
Ta	73 (27.4%)	36 (27.1%)	37 (27.8%)	
T1	193 (72.6%)	97 (72.9%)	96 (72.2%)	
**Tumor Grade**				>0.9
LG	20 (7.5%)	10 (7.5%)	10 (7.5%)	
HG	246 (92.5%)	123 (92.5%)	123 (92.5%)	
**Concomitant Cis**	25 (9.4%)	10 (7.5%)	15 (11.3%)	0.3
**Previous TURBT**	147 (55.3%)	75 (56.4%)	72 (54.1%)	0.7
**Re-TUR**	138 (51.9%)	63 (47.4%)	75 (56.4%)	0.14
**BCG schedule**				0.19
Only induction	34 (12.8%)	21 (15.8%)	13 (9.8%)	
Maintenance	232 (87.2%)	112 (84.2%)	120 (90%)	

^1^ Median (IQR); *n* (%). ^2^ Wilcoxon rank-sum test, Pearson’s Chi-square test.

## Data Availability

The data presented in this study are available on request from the corresponding author. The data are not publicly available.

## Supplementary Materials

The following supporting information can be downloaded at: https://www.mdpi.com/article/10.3390/cancers14040887/s1, Table S1: Baseline demographic and clinic-pathologic characteristics of the overall study population before propensity-score matching., Table S2: Maintenance BCG schedule and tolerability profile of the whole study population according to BCG strain (TICE vs. RIVM).
