# Supplementary material for: Compared Efficacy of Adjuvant Intravesical BCG-TICE vs. BCG-RIVM for High-Risk Non-Muscle Invasive Bladder Cancer (NMIBC): A Propensity Score Matched Analysis
Source: Cancers (Basel). 2022 Feb 10;14(4):887. doi: 10.3390/cancers14040887 (PMC8869903; doi:10.3390/cancers14040887)
Supplement: Supplementary file 1 [file cancers-14-00887-s001.zip › cancers-1567390-supplementary.pdf]

cancers

MDPI

Supplementary Materials: Compared Efficacy of Adjuvant Intravesical BCG-TICE vs. BCG-RIVM for High-Risk Non-Muscle Invasive Bladder Cancer (NMIBC): A Propensity Score Matched Analysis

Francesco Del Giudice, Rocco Simone Flammia, Benjamin I. Chung, Marco Moschini, Benjamin Pradere, Andrea Mari, Francesco Soria, Simone Albisinni, Wojciech Krajewski, Tomasz Szydełko, Ekaterina Laukhtina, David D'Andrea, Andrea Gallioli, Laura S. Mertens, Martina Maggi, Alessandro Sciarra, Stefano Salciccia, Matteo Ferro, Carlo Maria Scornajenghi, Vincenzo Asero, Susanna Cattarino, Mario De Angelis, Giovanni E. Cacciamani, Riccardo Autorino, Savio Domenico Pandolfo, Ugo Giovanni Falagario, Nicola D'Altilia, Vito Mancini, Marco Chirico, Francesco Cinelli, Carlo Bettocchi, Luigi Cormio, Giuseppe Carrieri, Ettore De Berardinis, Gian Maria Busetto and on behalf of European Association of Urology (EAU)—Young Academic Urologists (YAU) Urothelial Cancer Working Party

Table S1. Baseline demographic and clinic-pathologic characteristics of the overall study population before propensity-score matching.

Variable	Overall, *n* = 852
**Age,** years (IQR)	71 (64–79)
**Gender**	
Male	705 (82.7%)
Female	147 (17.3%)
**Smoking Status**	
No smoker	325 (38.1%)
Former smoker	305 (35.8%)
Active smoker	222 (26.1%)
**Tumor Size**	
<3 cm	598 (70.3%)
≥3 cm	253 (29.7%)
**Tumor focality**	
Unifocal	406 (47.7%)
Multifocal	446 (52.3%)
**T stage**	
Ta	262 (30.7%)
T1	590 (69.3%)
**Tumor Grade**	
LG	23 (2.7%)
HG	829 (97.3%)
**Concomitant CIS**	52 (6.1%)
**Previous TURBT**	342 (40.1%)
**Re-TUR**	445 (52.2%)
**BCG schedule**	
Only induction	447 (52.5%)
Maintenance	405 (47.5%)
**BCG strain**	
RIVM	719 (84.4%)
TICE	133 (15.6%)

Table S2. Maintenance BCG schedule and tolerability profile of the whole study population according to BCG strain (TICE vs RIVM).

Variable	BCG RIVM, *n* = 719	%	BCG TICE, *n* = 133	%	*p* Value
**Tolerability Profile:**					0.83
local toxicity	77	10.7	18	13.3	
systemic toxicity	35	4.8	7	5.2	
local and systemic toxicity	59	8.2	10	7.8	
drop-off due to side effects	38	5.3	6	4.5	
**BCG dosage:**					0.56
full dose	672	93.5	121	91	
half dose	37	5.1	9	6.8	
one-third	10	1.4	3	2.2	
**Duration of maintenance*:**					0.77
completed 3-yr schedule	28	9.8	14	11.7	
completed 1-yr schedule	157	55.2	62	51.7	
maintenance for < 1-yr	100	35	44	36.6	
**Number of instillations*:**					0.97
7–15	49	17.2	22	18.2	
16-21	183	64.3	77	63.9	
≥22	53	18.5	21	17.9	

* Numbers and percentages referred to only patients on maintenance schedule.
